# Screening patients in general practice for advanced chronic liver disease using an innovative IT solution: The Liver Toolkit

**DOI:** 10.1097/HC9.0000000000000482

**Published:** 2024-06-27

**Authors:** David S. Prince, Shakira Hoque, Christy Kim, Salim Maher, Jane Miller, Phoebe Chomley, Janice Pritchard-Jones, Sally Spruce, Nathan McGarry, David Baker, Penelope Elix, Ken Liu, Simone I. Strasser, Brendan Goodger, Amany Zekry, Geoffrey W. McCaughan

**Affiliations:** 1 AW Morrow Gastroenterology and Liver Centre, Royal Prince Alfred Hospital, Sydney, New South Wales, Australia; 2 Liver Injury and Cancer Program, Centenary Institute, Sydney, New South Wales, Australia; 3 Department of Gastroenterology and Liver, Liverpool Hospital, Liverpool, New South Wales, Australia; 4 Faculty of Medicine and Health, The University of New South Wales, Sydney, New South Wales, Australia; 5 Department of Gastroenterology and Hepatology, St George Hospital, Kogarah, New South Wales, Australia; 6 Central and Eastern Sydney Primary Health Network, Mascot, New South Wales, Australia; 7 East Sydney Doctors Darlinghurst, New South Wales, Australia; 8 Fountain Street General Practice, Alexandria, New South Wales, Australia; 9 Faculty of Medicine and Health, University of Sydney, Sydney, New South Wales, Australia

## Abstract

**Background:**

Identifying patients with undiagnosed advanced chronic liver disease (ACLD) is a public health challenge. Patients with advanced fibrosis or compensated cirrhosis have much better outcomes than those with decompensated disease and may be eligible for interventions to prevent disease progression.

**Methods:**

A cloud-based software solution (“the Liver Toolkit”) was developed to access primary care practice software to identify patients at risk of ACLD. Clinical history and laboratory results were extracted to calculate aspartate aminotransferase-to-platelet ratio index and fibrosis 4 scores. Patients identified were recalled for assessment, including Liver Stiffness Measurement (LSM) via transient elastography. Those with an existing diagnosis of cirrhosis were excluded.

**Results:**

Existing laboratory results of more than 32,000 adults across nine general practices were assessed to identify 703 patients at increased risk of ACLD (2.2% of the cohort). One hundred seventy-nine patients (26%) were successfully recalled, and 23/179 (13%) were identified to have ACLD (LSM ≥10.0 kPa) (10% found at indeterminate risk [LSM 8.0–9.9 kPa] and 77% low risk of fibrosis [LSM <8.0 kPa]). In most cases, the diagnosis of liver disease was new, with the most common etiology being metabolic dysfunction–associated steatotic liver disease (n=20, 83%). Aspartate aminotransferase-to-platelet ratio index ≥1.0 and fibrosis 4 ≥3.25 had a positive predictive value for detecting ACLD of 19% and 24%, respectively. Patients who did not attend recall had markers of more severe disease with a higher median aspartate aminotransferase-to-platelet ratio index score (0.57 vs. 0.46, *p*=0.041).

**Conclusions:**

This novel information technology system successfully screened a large primary care cohort using existing laboratory results to identify patients at increased risk ACLD. More than 1 in 5 patients recalled were found to have liver disease requiring specialist follow-up.

## Graphical abstract

**Figure:**
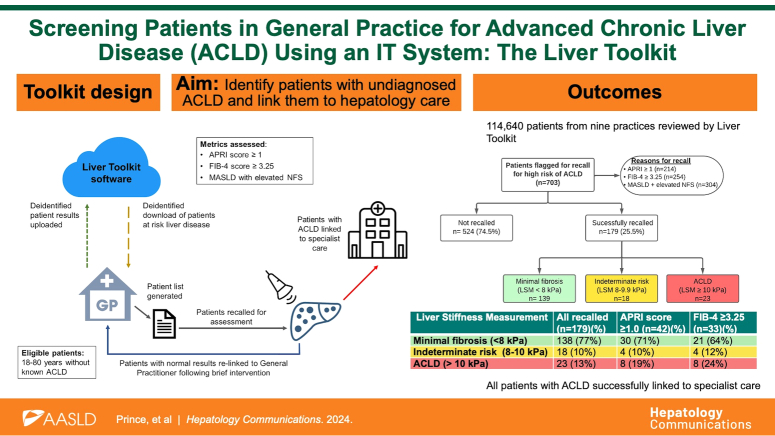

## INTRODUCTION

More than 1.5 billion people around the world are living with chronic liver disease with the majority being undiagnosed.^1^ Globally, cirrhosis leads to more than 1.3 million deaths annually and is responsible for 3.5% of all-cause mortality.^1,2^ Detecting patients with advanced chronic liver disease (ACLD) is a major current public health challenge. Patients with compensated ACLD have minimal symptoms and a good overall prognosis,^3^ and early detection of patients can allow for disease-specific treatment that can lead to fibrosis regression and resolution of portal hypertension.^4,5^ Unfortunately, the majority of patients are only diagnosed when they exhibit features of end-stage decompensated disease, which has a high rate of mortality without liver transplantation.^6^ Thus, there is an urgent need to change the current paradigm for diagnosing chronic liver disease. This has been recognized by the European Union in their new endeavor called the LiverScreen project (not yet undertaken), which has similarities to the study reported in our paper.^7^

Over the last 2 decades, several serum noninvasive tests (NITs) have been developed to identify patients with liver fibrosis. These tests have predominately been validated in patients with known liver disease in specialist care settings and are reported to have good diagnostic accuracy.^8–10^ Over time, the best thresholds for these tests have changed, and the literature in this area is still in flux. Less work has been done validating these tests in primary care settings or in developing novel, innovative models of care which integrate them into routine clinical practice to allow generalized screening of at-risk populations.

There has been an exponential rise in the testing of liver biochemistry in primary care, with most of these investigations being ordered for other clinical reasons.^11^ As many as 20% of these tests may be abnormal, presenting a significant burden for primary care physicians, and up to 50% of results receive no follow-up.^11^ However, it is known that even minor abnormalities in liver function biochemistry can be associated with a higher risk of long-term mortality.^12^ Identifying patients in primary care who will benefit the most from specialist referral for further assessment remains a challenge, and there is debate about the best approach.^13^

This study evaluates the use of a novel information technology cloud-based software solution, which accesses medical records of general practices and uses existing clinical information and laboratory results to identify patients who are at increased risk of ACLD. The aim of this study is to assess if this approach is effective in detecting patients with undiagnosed ACLD and linking them to specialist care.

## METHODS

### Liver Toolkit development

The Liver Toolkit is a cloud-based software platform that was developed by a medical software company (Outcome Health) as a pilot instrument specifically for this project. This tool was designed in collaboration with staff from the Central and Eastern Sydney Primary Health Network (CESPHN), Sydney and South Eastern Sydney Local Health Districts, and community general practices (sometimes referred to as primary care practices).

The tool was designed to analyze general practice electronic medical records to identify patients at increased risk of ACLD. The tool accessed both clinical information and investigation results stored with General Practitioner (GP) practice software. Clinical parameters assessed included previous diagnoses of viral hepatitis infection, alcohol use disorder or excess alcohol consumption, and hepatic steatosis (previously known as NAFLD). Laboratory test results from the last 2 years were accessed to calculate the aspartate aminotransferase-to-platelet ratio index (APRI)^14^, fibrosis 4 (FIB-4)^15^ score, and NAFLD fibrosis score (NFS).^16^ For patients with multiple blood tests within the last 2 years, the most recent results were used to calculate these noninvasive metrics.

**FIGURE 1:**
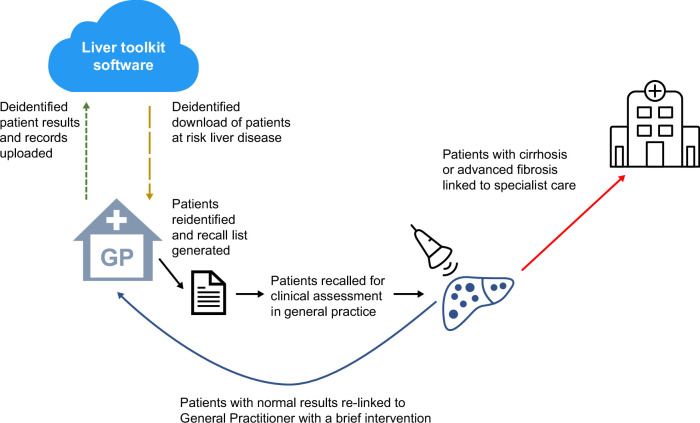
Liver Toolkit model. Abbreviation: GP, General Practitioner.

**FIGURE 2:**
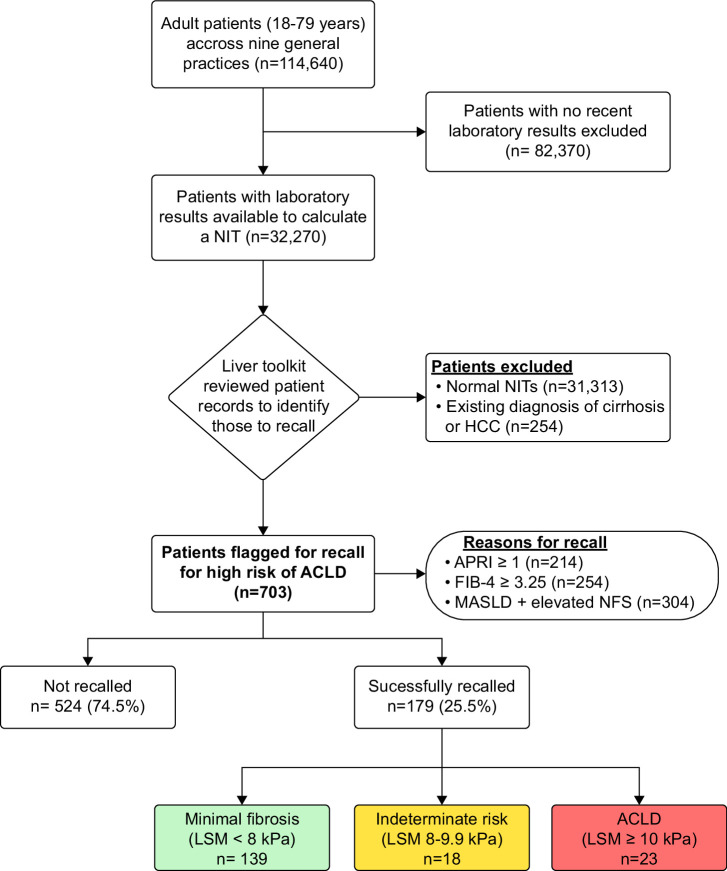
Liver Toolkit patient flow diagram. Abbreviations: ACLD, advanced chronic liver disease; APRI, aspartate aminotransferase-to-platelet ratio index, FIB-4, fibrosis 4 score; LSM, liver stiffness measurement, MASLD, metabolic dysfunction–associated steatotic liver disease, NFS, NAFLD fibrosis score, NIT, noninvasive test.

The Liver Toolkit was designed to remotely review the medical records from consenting practices. All data transferred to the Liver Toolkit platform were in a deidentified format. A list of at-risk patients identified by the Liver Toolkit was only downloadable inside each general practice by project staff (Figures 1, 2). The tool underwent comprehensive beta-testing with simulated patient data prior to implementation.

### Practice enrollment

Suitable general practices were identified by the CESPHN. To be eligible, practices required appropriate practice software (Medical Director or Best Practice), which allowed integration of the digital Liver Toolkit. Practices were contacted by the CESPHN and offered to participate, and those who agreed signed written consent on behalf of their medical practice. The CESPHN attempted to engage practices in a range of different geographic areas across their catchment area.

### Inclusion criteria

The Liver Toolkit assessed adult patients between 18 and 79 years of age. This group was chosen pragmatically to include patients who would benefit from the detection of undiagnosed ACLD while minimizing potential harm from recalling older patients who would be less likely to benefit.

### Exclusion criteria

Patients were excluded from the project if they:

1. Were outside the above age range,
2. Had not been seen by GP in the last 2 years or,
3. Had a known diagnosis of cirrhosis or HCC

### Recall criteria

Patients were flagged for recall by the liver toolkit if they met either of the following criteria:

1. APRI score ≥1 and/or FIB-4 score ≥3.25,
2. An existing diagnosis of metabolic dysfunction–associated steatotic liver disease (MASLD) and an elevated NFS (> −1.455 for those 65 or less and >0.12 for those older than 65 y of age).

These thresholds were developed based on previous studies demonstrating good sensitivity for the detection of compensated ACLD.^17^ The recall criteria are outlined further in Table 1.

**TABLE 1:** Noninvasive tests assessed using the Liver Toolkit

Noninvasive test (NIT)	Components	Initial validation cohort	Patients assessed	No. patients evaluated with NIT by liver toolkit software	Threshold used for recall by liver toolkit
Fibrosis 4 score (FIB-4)	Age, AST, ALT, platelet count	HIV and HCV coinfection^15^	18–79 y with relevant laboratory results	31,199	≥3.25
AST-to-platelet ratio (APRI)	AST, platelet count	HCV^14^	18–79 y with relevant laboratory results	32,270	≥1.0
NAFLD fibrosis score (NFS)	Age, hyperglycemia, BMI, platelet count, albumin, AST/ALT ratio	NAFLD^16^	Patients with MASLD	2, 632^a^	Age ≤65: Threshold > −1.455 Age >65 y: Threshold > 0.12

a Lower number of patients assessed using the NFS due to required variables to calculate the (BMI, hyperglycemia) not be available in all patients.

Abbreviations: ALT, alanine transferase; APRI, aspartate aminotransferase-to-platelet ratio; AST, aspartate aminotransferase; BMI, body mass index; FIB-4, fibrosis 4 score; MASLD, metabolic dysfunction–associated steatotic liver disease; NIT, noninvasive test; NFS, NAFLD fibrosis score.

### Recall process

A list of patients identified based on the above criteria was given to each general practice for recall (see Supplemental Figure S1 for example list, Supplemental Digital Content 1, http://links.lww.com/HC9/A949). Treating clinicians reviewed this list and could exclude patients if they were deemed inappropriate for recall (significant medical comorbidities making further assessment futile or a clear alternative explanation of their abnormal result). This was left up to the discretion of the primary care specialist.

Practice staff were asked to contact patients directly to inform them of the project and invite them to attend a recall visit. Practices were provided with proforma materials for recall letters, emails, and text messages developed in consultation with a consumer representative from Hepatitis New South Wales (a state-based community organization). Practices were requested to contact patients at least 3 times using a range of methods (telephone calls, letters, or text messages), and if recall was not possible, to record a reason why.

### Direct referral

Throughout the project, GPs were also allowed to directly refer other patients not on the recall list for assessment by the Liver Toolkit team if they had clinical concerns for undiagnosed liver disease.

### Clinical assessment

Patients successfully recalled underwent clinical assessment with the Liver Toolkit team (comprised of a hepatology nurse and gastroenterology doctor). The preferred model for this was as an outreach service within the general practice to reduce complexity for patients and maintain the relationship with their general practice. Patients were also able to visit the hospital outpatient clinic as an alternative model.

The recall visit included assessment of body mass index, alcohol intake and risk factors for liver disease and a physical examination, including transient elastography (TE) using FibroScan (Echosens, Paris, France). Alcohol intake was qualified in standard drinks per week (with 1 standard drink defined at 10 g of ethanol).^18^ TE was conducted using the standard approach to obtain a liver stiffness measurement (LSM) using the appropriate probe type for each patient (either M or XL). TE assessments required a minimum of 10 measurements with a success rate of ≥60% and an IQR of ≤30% (if LSM ≥7) were deemed valid.^19,20^

### Assessment of liver disease severity at recall

All patients recalled underwent a brief intervention designed to increase awareness of liver health and modifiable risk factors for liver disease. Following TE assessment, patients with a normal LSM or a result suggestive of low risk of significant fibrosis (<8.0 kPa) were returned to their GP for ongoing care. Patients with an indeterminate of level of fibrosis (8.0–9.9 kPa) or high risk of ACLD (defined as an LSM ≥10.0 kPa)^21^ were recommended to undergo a comprehensive liver screen and further follow-up in secondary care. There, LSM thresholds were developed based on the results of a previous meta-analysis.^10^ Cirrhosis was defined as an LSM ≥13.0 with radiological and/or biochemical evidence of cirrhosis as assessed by hepatology. HCC surveillance was commenced in patients diagnosed with cirrhosis.

### Outcomes

The primary outcomes for this study were the proportion of patients successfully recalled and assessed via this novel Liver Toolkit model and the proportion of patients identified with ACLD (LSM ≥10.0 kPa). Secondary outcomes included the number of TE visits conducted, the number of referrals to secondary care, and the overall utility of this approach in detecting undiagnosed liver disease. Staff time to perform recall assessments was also recorded throughout the project.

### Comparison to patients who did not attend recall

Patients who did not attend recall were compared to those who did attend to assess for differences in demographics or laboratory results. For this purpose, historical data stored with GP practice software on non-recalled patients was used. In some, this was incomplete.

### Ethics and data management

Study data were collected and stored using the REDCap electronic data capture tool hosted by the Sydney Local Health District. All analyses were performed using SPSS (version 27.0, Armonk, NY). Continuous variables were summarized as means with SDs or medians with IQR as appropriate. Comparisons between groups were performed using chi-squared or Fisher exact test for categorical variables and the Student *t* test or Mann-Whitney *U* test as appropriate for continuous variables. A *p*-value <0.05 was considered statistically significant. The study was conducted according to the Declaration of Helsinki and was approved by the Sydney Local Health District Human Research Ethics Committee (2019/ETH13364). Written informed consent was obtained from all general practices involved in the study and from all patients successfully recalled.

### MASLD nomenclature

This study was designed and conducted prior to a recent change in nomenclature recommended by major hepatology societies.^22^ As such, the study protocol initially referred to NAFLD. In an attempt to prevent confusion and adopt the new terminology, MASLD has been used where possible to refer to patients with hepatic steatosis and cardiometabolic risk factors. Hepatic steatosis has been used to refer to patients with a previously coded diagnosis of NAFLD, where it is not clear if they satisfy the new MASLD criteria.

## RESULTS

### Practice recruitment

Fifteen practices were recruited for the Liver Toolkit project, and study ran for a 27-month period (October 2020 to December 2022). Two practices were excluded shortly after consent due to software incompatibility between the Liver Toolkit and the practice software (Zedmed). Four other practices withdrew from the project during the COVID-19 pandemic. The remaining 9 practices had a total of 114,640 active adult patients at baseline with a median number of patients of 10,878 (IQR: 7400–14,664) (Table 2). Liver biochemistry and full blood count results were available to calculate APRI and FIB-4 scores for 32,270 and 31,199 patients, respectively (28.1% and 27.2% of the active cohort), (Figure 2). A total of 2632 patients with an existing diagnosis of MASLD were assessed using the additional NFS criterion.

**TABLE 2:** Adult patient numbers and Liver Toolkit recall rates by general practice

Practice	Active adult patient number	Patients with laboratory results to calculate at least 1 NIT (% active), n (%)	Patients with a pre-existing MASLD diagnosis	Patients flagged for recall by liver toolkit	Number successfully recalled, n (%)	Other patients referred directly by GP for assessment
1	10,878	3062 (28.1)	208	76	26 (34)	8
2	4695	2,504 (53.3)	403	95	37 (39)	13
3	10,921	4,811 (44.1)	207	160	34 (21)	0
4	7400	3605 (48.7)	167	60	10 (17)	1
5	8920	2830 (31.7)	270	67	23 (34)	3
6	31,545	5614 (17.8)	544	67	22 (33)	1
7	22,348	5473 (24.5)	660	112	11 (10)	0
8	14,664	3712 (25.3)	124	59	15 (25)	0
9	3269	659 (20.2)	51	7	1 (14)	2
Total	114,640	32,270 (28.1)	2634	703	179 (26)	28

Abbreviations: FIB-4, fibrosis 4 score; GP, General Practitioner; MASLD, metabolic dysfunction–associated steatotic liver disease; NIT, noninvasive test.

### Patients flagged by the Liver Toolkit

There were 703 patients identified for recall (Figure 2). Reasons for recall were APRI ≥1 (n=214), FIB-4 ≥3.25 (n=254), and MASLD with an elevated NFS (n=304) (69 patients had both an elevated APRI and FIB-4 score). The median number of patients identified for recall per practice was 67 (IQR: 60–95). Twenty-eight practice recall visits were conducted by the Liver Toolkit team between November 2020 and December 2022. The project was initially scheduled to finish in June 2022 but was extended due to interruptions related to the COVID-19 pandemic. A total of 225.5 hours were spent in general practices by project staff as part of the recall (consisting of 116, 62, and 47.5 hours by medical staff, TE technicians, and nursing staff, respectively).

Overall, 179 patients were successfully recalled (25.5% of the recall cohort); however, there was variation between practices, with the rate of successful recall ranging from 9.8% to 38.9%. Recall response rates based on indication for recall were 19.6% (42/214), 13.0% (33/254), and 38.5% (117/304) for an elevated APRI, elevated FIB-4, and MASLD with an elevated NFS, respectively. This latter group of patients was more likely to respond to recall than those with an elevated APRI or FIB-4 (*p*<0.0001, χ^2^=33.69).

The majority of recall visits were conducted in general practice (n=165, 92%), with only 14 patients (8%) preferring to visit the hospital for assessment. A further 28 patients were directly referred by their GP to the project team (not based on the Liver Toolkit) and were assessed through the project. All patients consented to participate in the project analysis. No complaints or negative feedback were received from participants in the project.

### Patient characteristics

Patients successfully recalled were mostly male (59%) with a mean age of 62.0 years (SD ±10.5) and a median body mass index of 28.1 kg/m^2^ (IQR: 25.0–32.6) (Table 3). A diagnosis of MASLD, dyslipidemia, and diabetes/impaired glucose was present in 74% (n=132), 51% (n=92), and 39% (n=69), respectively. Patients referred directly to the project team by their GP for assessment had similar overall characteristics with no differences in age, gender, weight, alcohol use, or prevalence of dyslipidemia or diabetes compared to patients identified by the Liver Toolkit. Patients directly referred by their GP were less likely to have a diagnosis of MASLD (*p*<0.01) and more likely to have a diagnosis of chronic hepatitis B (*p*<0.01) compared with Liver Toolkit-identified patients. Patients referred directly by their GP also had higher median platelet counts and albumin levels (193 vs. 242×10^9^/L, *p*=0.002 and 43 vs. 46 g/L, *p*=0.020, respectively).

**TABLE 3:** Characteristics of patients assessed as part of liver toolkit project

	Liver toolkit patients (n=179)(%)	GP direct referral (n=28)(%)	*p*
Age, years (mean ±SD)	62.0 (±10.54)	59.1 (±16.44)	0.373
Gender	Male=105 (58.7) Female=73 (40.8) Transgender=1 (0.6)	Male=16 (57.1) Female=12 (42.9)	0.854
Country of birth	Australia—83 China—60 Vietnam—5 Macedonia—3 Other—28	Australia—14 China—13 Vietnam—1	—
Weight, kg (median [IQR])	80.2 (68.8–97.1)	74.2 (62.2–87.0)	0.087
Height, cm (median [IQR])	168 (160–178)	169 (158–174)	0.389
BMI, kg/m^2^ (median [IQR])	28.1 (25.0–32.6)	26.4 (22.7–32.1)	0.246
Hepatic steatosis	132 (73.7)	13 (46.4)	**<0.01**
Diabetes or impaired glucose tolerance (IGT)	Diabetes—54 (30.2) IGT—15 (8.4)	Diabetes—7 (25.0) IGT—1 (3.6)	0.310
Dyslipidemia	92 (51.4)	11 (39.3)	0.402
Standard drinks per week (median [IQR])^a^	1 (0–10)	0 (0–12.5)	0.563
Known chronic hepatitis B	8 (4.5)	11 (39.3)	**<0.01**
Known chronic hepatitis C	5 (2.8)	0	0.747
AST, U/L (median [IQR])	32 (24–44)	28 (23–41)	0.308
ALT, U/L (median [IQR])	34 (24–59)	33 (24–39)	0.519
Platelet count, 10^9^/L (median [IQR])	193 (162–228)	243 (193–275)	**0.002**
Bilirubin, μmol/L (median [IQR])	13 (10–17)	14 (9–17)	0.552
Albumin. g/L (median [IQR])	43 (41–46)	46 (43–47)	**0.020**
Creatinine, μmol/L (median [IQR])	73 (64–85)	75 (70–80)	0.471
Reason for recall	Elevated APRI=42 Elevated FIB-4=33 High risk NAFLD=117	GP referral – 28	—

a Data missing in 25 patients.

Bold values indicate significance *p*<0.05.

Abbreviations: ALT, alanine transaminase; APRI, aspartate aminotransferase-to-platelet ratio index; AST, aspartate aminotransferase; BMI, body mass index; FIB-4, fibrosis 4 score; GP, General Practitioner; IGT, impaired glucose tolerance.

### Elastography results

All patients recalled from the Liver Toolkit underwent LSM using TE with no unsuccessful or invalid results. The median LSM was 5.9 kPa (IQR: 4.4–7.9) (Table 4). ACLD (LSM ≥10.0) was identified in 23 patients recalled (12.8%) (15 of which were determined to have cirrhosis). A further 18 (10.1%) had an indeterminate result (LSM 8.0–9.9 kPa), requiring further evaluation. The majority of patients (n=138, 77.1%) had an LSM result indicative of low risk of fibrosis (including 94 [52.5%] with a normal LSM).

**TABLE 4:** Transient elastography results by patient type and reason for recall

Transient elastography result (kPa)	Liver Toolkit patients (n=179), n (%)	Direct GP referral (n=28), n (%)	High APRI score (≥1.0) (n=42), n (%)	High FIB-4 score (≥3.25) (n=33), n (%)	Both high APRI and FIB-4 (n=20), n (%)	MASLD (n=117), n (%)
Low risk of significant fibrosis (LSM <8.0)	138 (77.1)	27 (96.4)	30(71.4)	21 (63.6)	12 (60.0)	91 (77.8)
Indeterminate risk (LSM: 8.0–9.9)	18 (10.1)	0	4 (9.5)	4 (12.1)	3 (15.0)	13 (11.1)
High-risk ACLD (LSM ≥10.0)	23 (12.8)	1 (3.6)	8 (19.0)	8 (24.2)	5 (25.0)	13 (11.1)

Abbreviations: ACLD, advanced chronic liver disease; APRI, aspartate aminotransferase-to-platelet ratio index; FIB-4, fibrosis 4 score; GP, General Practitioner; kPa, kilopascal; LSM, liver stiffness measurement; MASLD, metabolic dysfunction–associated steatotic liver disease.

Among the cohort referred directly by their GP for assessment, only 1 had ACLD, with 27/28 (96.4%) having a LSM suggestive of low fibrosis risk. Patients identified by the Liver Toolkit were significantly more likely to have ACLD or an indeterminate result requiring ongoing follow-up compared to those directly referred by their GP (22.9% vs. 3.6%, *p*=0.021). There were no significant differences in the rates of ACLD detection between patients recalled based on an elevated APRI, FIB-4, or MASLD with an elevated NFS.

### Characteristics of patients found to have significant liver disease

Among patients found to have ACLD (n=23), MASLD was the most common etiology (n=20, 83%) followed by alcohol (n=5, 20%) (Table 5). In most cases, the patient was unaware of the diagnosis of liver disease (83%) and had never seen a gastroenterologist (79%). Concerningly, 5 patients with cirrhosis detected through the project had evidence of portal hypertension, and 2 had decompensated disease (both Child-Pugh B).

**TABLE 5:** Characteristics of patients identified with advanced chronic liver disease or indeterminate risk of fibrosis

	Advanced chronic liver disease (n=23), n (%) ^a^	Indeterminate risk (n=18), n (%) ^a^
Male gender	18 (78)	13 (72)
Age, y (mean ±SD)	62.7 (±9.7)	67.2 (±7.7)
BMI, kg/m^2^ (median [IQR])	33.3 (29.7–38.9)	28.9 (26.4–34.6)
Diabetes or impaired glucose tolerance	14 (61)	9 (50)
Dyslipidemia	10 (43)	11 (61)
Etiology of liver disease^b^		
MASLD	20 (87)	12 (67)
Alcohol	5 (22)	3 (17)
Hepatitis B	1 (4)	2 (22)
Hepatitis C	1 (4)	1 (11)
Previously seen a gastroenterologist	5 (22)	2 (11)
New diagnosis of liver disease	19 (83)	16 (89)
LSM (kPa) (median [IQR])	15.4 (11.5–19.5)	8.8 (8.2–9.2)
Cirrhosis^c^	15 (65)	NA
Portal hypertension	5	—
Ascites	2	—
Encephalopathy	1	—
Child-Pugh Score	5=11, 6=2, 7=1, 9=1	—
AST, U/L (median [IQR])	43 (32–58)	36 (23–45)
ALT, U/L (median (IQR))	54 (28–66)	34 (23–65)
Platelet count, 10^9^/L (median [IQR])	160 (140–247)	177 (161–217)
Bilirubin, umol/L (median [IQR])	11 (11–28)	14 (10–20)
Albumin, g/L (median [IQR])	42 (39–44)	42 (38–45)
Creatinine, μmol/L (median [IQR])	75 (64–96)	75 (68–83)
Liver ultrasound in last 6 mo	5 (22)	5 (28)

a Advanced Chronic Liver Disease: LSM ≥10 kPa; indeterminate fibrosis: LSM=8-9.9 kPa.

b Multiple etiologies of liver disease possible.

c Cirrhosis: Defined by LSM ≥13.0 kPa with radiological and/or biochemical features of cirrhosis and assessed by hepatologist.

Abbreviations: ALT, alanine transaminase; AST, aspartate aminotransferase; BMI, body mass index; FIB-4, fibrosis 4 score; kPa, kilopascal; LSM, liver stiffness measurement; MASLD, metabolic dysfunction–associated steatotic liver disease; NA, not applicable.

### APRI and FIB-4 operating characteristics

The positive predictive value (PPV) for APRI ≥1.0 and FIB-4 ≥3.25 for detecting ACLD were 20% (8/42) and 24% (8/33), respectively. In all patients with an elevated APRI or FIB-4, a repeat test was arranged where possible. For the patients with an elevated APRI, 28/42 (67%) continued to have a result ≥1 on subsequent testing, and no patient who had normalization of APRI on repeat testing was found to be ACLD. For patients with an elevated FIB-4, 22/33 (67%) continued to have a result ≥3.25 on repeat testing. The PPV of APRI ≥1.0 and FIB-4 ≥3.25 for detecting ACLD rose to 43% (12/28 patients) and 40% (9/22) when patients with 2 consecutive abnormal results were considered (Supplemental Table S1, Supplemental Digital Content 1, http://links.lww.com/HC9/A949).

### Patients who did not attend recall

Baseline demographic and laboratory results were available for a cohort of 320 patients from the Sydney Local Health District who did not attend recall (representing 61% [320/524] of all nonattendees) (Table 6). Compared to Liver Toolkit patients who attended recall, nonparticipants were younger (58.6 vs. 62.0 y, *p*=0.001) and more likely to be male (69% vs. 59%, *p*=0.021). Nonparticipants had higher median aspartate aminotransferase and alanine transferase results (39 vs. 32 U/L, *p*<0.001 and 44 vs. 33 U/L, *p*<0.001, respectively) and were more likely to have an APRI score ≥1.0 (34% vs. 25%, *p*=0.032). Nonattendees were less likely to have a diagnosis of hepatic steatosis or dyslipidemia (54% vs. 74%, *p*<0.001 and 20% vs. 51%, *p*<0.001, respectively).

**TABLE 6:** Comparison of patients successfully recalled compared to sample of those not recalled

	Liver Toolkit patients successful recalled (n=179) (%)	Patients not recalled^a^ (n=320) (%)	*p*
Age, years (mean ±SD)	62.0 (±10.54)	58.6 (±13.16)	**0.001**
Gender, n (%)	Male=105 (58.7) Female=73 (40.8) Transgender=1 (0.6)	Male=221 (69.0) Female=98 (30.6) Transgender=1 (0.1)	**0.021**
APRI score (median [IQR])	0.46 (0.31–0.99)	0.57 (0.34–1.15)	**0.041**
FIB-4 score (median [IQR])	1.81 (1.41–3.03)	1.90 (1.36–2.99)	0.805
NFS (median [IQR])	−0.52 (−1.15-0.55)	−0.83 (−1.47 to 0.2)	**0.006**
BMI, kg/m^2^ (median [IQR])^b^	28.1 (25.0–32.6)	29.4 (24.6–32.9)	0.832
Hepatis steatosis, n (%)	132 (73.7)	174 (54.4)	**<0.001**
Diabetes diagnosis, n (%)	54 (30.2)	76 (23.8)	0.209
Dyslipidemia, n (%)	92 (51.4)	64 (20.0)	**<0.001**
Standard drinks of alcohol per week, n (%)^b^	—	—	0.378
Nondrinker	76 (42.5)	100 (31.2)	—
1–10	40 (22.3)	57 (17.)	—
11–20	16 (8.9)	21 (6.6)	—
21–30	5 (2.8)	11 (3.4)	—
>30	17 (9.5)	28 (8.8)	—
Unknown	25 (14.0)	103 (32.2)	—
Known hepatitis B, n (%)	8 (1.1)	18 (5.6)	0.677
Known hepatitis C	5 (2.8)	16 (5.0)	0.158
AST, U/L (median [IQR])	32 (24–44)	39 (27-78)	**<0.001**
ALT, U/L (median [IQR])	34 (24–59)	44 (27-80)	**<0.001**
Platelet count, 10^9^/L (median [IQR])	193 (162–228)	201 (167-235)	0.313
Bilirubin, umol/L (median [IQR])	13 (10–17)	13 (10-16)	0.316
Albumin, g/L (median [IQR])	43 (41–46)	43 (40-45)	0.067
INR (median [IQR])	1.0 (1.0–1.0)	1.0 (1.0–1.1)	0.255
Percentage with APRI ≥1	24.6% (42/171)	33.5% (92/275)	**0.032**
Percentage with FIB-4 ≥3.25	21.0% (34/162)	21.1% (58/275)	0.980

a Data available for 320/524 patients not recalled.

b BMI and alcohol intake assessed at appointment in recall group versus GP record for patients not recalled.

Bold values indicate significance *p*<0.05.

Abbreviations: ALT, alanine transaminase; APRI, aspartate aminotransferase-to-platelet ratio index; AST, aspartate aminotransferase; BMI, body mass index; FIB-4, fibrosis 4 score; GP, General Practitioner; kPa, kilopascal; NFS, NAFLD fibrosis score.

## DISCUSSION

This novel program, the first of its kind in Australia, used an information technology solution (the Liver Toolkit) to screen existing data stored within general practice medical records to identify patients with undiagnosed ACLD. Blood tests from over 32,000 adult patients were analyzed to identify 703 individuals at high risk of ACLD (~2.2% of the cohort). However, only 25% of these patients were successfully recalled for further assessment, and only 1 in 5 patients were confirmed to have either ACLD or an indeterminate result requiring further evaluation. This study highlights the complexities and limitations of population screening for ACLD.

The Liver Toolkit model used pre-existing readily available data and leveraged electronic practice software to identify patients at risk of liver disease. The tool did not interfere with frontline health care by GPs. unlike other models that require active GP recruitment of patients at the time of clinical consultation (resulting in poor uptake).^11^ Patients and GPs anecdotally reported satisfaction with this approach as it maintained the patient’s established relationship with their primary care specialist. The Liver toolkit assessed three blood-based nonpatented NITs for the detection of ACLD. NIT selection was based on available literature at the time the project was designed. It should be acknowledged there are other NITs that were not assessed as part of this study, such as the BARD score (BMI, AST/ALT ratio and diabetes)^23^, aspartate aminotransferase/alanine transferase ratio, and the newer Agile 3+ score.^24^

The Liver Toolkit compares favorably to other similar studies. In a large German cohort study, ~11,000 patients were screened, and those with an APRI ≥0.5 (488 patients or 4.12% of this cohort) underwent further assessment with an overall ACLD detection rate of ~17%.^25^ Our model, with a more stringent APRI cutoff and incorporation of other indices (FIB-4 and NFS) was able to screen a larger volume of patients with a similar overall utility. A British study examined a liver referral pathway using the aspartate aminotransferase:alanine transferase ratio and clinical risk factors for liver disease (harmful alcohol or metabolic disease) and found a rate of abnormal LSM (≥8 kPa) requiring further assessment (22.9%) similar to our study.^26^ A recent Chinese and Malaysian study used a similar software approach with “pop-up messaging” for patients with elevated FIB-4 or APRI scores and found this was superior to the standard of care for linking patients to hepatology services for fibrosis assessment.^27^

Primary care approaches to detect significant liver disease in the community are now seen as crucial in order to decrease the burden of liver disease and introduce personalized strategies to prevent disease progression. A recent example of this is the publication in *Nature Medicine* of the planned LiverScreen project funded by the European Union’s Horizon Program.^7^ The overall aim of the program is to provide cost-effective screening for the early detection of liver fibrosis using NITs. The first phase plans to use blood biomarkers as well as TE in patients with known liver disease already under care. The planned second phase is much more like our Liver Toolkit project, where 10,000 persons without known liver disease, selected from the community, across 4 countries will undergo noninvasive blood tests followed by the confirmation of the presence of liver fibrosis in specialized hepatology services. Outcomes from this project are likely sometime away and it will be of interest to compare results to our Liver Toolkit project and similar other studies.

The overall precision of Liver Toolkit, however, was low, with >50% of patients assessed having an entirely normal result on TE. APRI ≥1.0 and FIB-4 ≥3.25 both had PPV for ACLD of <25%. This is much lower than has been previously reported in secondary care cohorts but similar to the rate found in a large population study.^28^ This highlights the difficulties with using these tests in the general population. This has been described as the “spectrum effect” where sensitivity and PPV fall in relation to the disease prevalence^29^. Additionally, when the NITs were repeated, APRI and FIB-4 scores normalized in one-third of patients suggesting in real-world usage, there may be significant day-to-day variability. This confirms previous work suggesting there may be a role for serial measurement of such indices.^30^

Interestingly, the Liver Toolkit process identified more patients with liver fibrosis than direct GP referrals during the same period. This may be partially explained by GPs being more likely to refer patients with viral hepatitis or abnormal liver function tests for management advice as opposed to screening for cirrhosis. However, only 3.6% of patients referred by their GP due to clinical concern had an LSM requiring further follow-up for ACLD compared to 22.9% of patients identified by the toolkit. This highlights the difficulties in detecting asymptomatic compensated ACLD in the general population and the possible utility of NITs using readily available parameters.

This project relied on TE to quantify the extent of liver fibrosis in patients flagged by blood-based indices. Currently, TE is the accepted reference NIT for detecting fibrosis due to its high accuracy and reproducibility^17,31,32^; however, it is not a perfect test, and liver biopsy continues to have a role in some cases. As demonstrated in our study, 18 patients (10% of the recall cohort) had an indeterminate result on TE. It is currently unclear whether such patients should have ongoing TE surveillance or proceed directly to liver biopsy. There is, however, evidence to suggest further evaluation can lead to changes in clinical management.^33^ The considerable indeterminate cohort makes more widespread population screening for liver disease potentially problematic.

The lack of a control group was a potential limitation of this project. As such, it is unknown what proportion of the 97.8% of adult patients not flagged for recall by the Liver Toolkit may have had a significant liver disease that was not recognized (ie, the false negative rate). This project, however, was designed with a pragmatic, real-world approach to attempt to stratify the very large number of patients in primary care with abnormal results to determine who would best benefit from a targeted liver intervention in the setting of finite health care resources.

Another limitation of the study was the relatively low response rate to recall (~25% overall). This is only slightly less than the 33% response rate achieved with the Australian National Bowel Cancer Screening Program, highlighting the potential difficulties in engaging patients in preventative screening for asymptomatic disease even when programs are evidence-based and well resourced.^34^ The COVID-19 pandemic may have also contributed to the low response rate, with the project being interrupted for 8 months. The rates of successful recall also differed significantly between practices (range 10%–40%), with higher-performing practices often having additional staff to assist with recall. Interestingly, the rates of recall uptake varied significantly between patients with an elevated APRI or FIB-4 and to those with MASLD. Finally, patients who did not respond to the recall had higher liver transaminases and APRI scores, suggesting that patients at the highest risk of underdiagnosed liver disease may have not been assessed. An independent qualitative analysis of the liver toolkit, including structured interviews of project staff and patients and quantification of resource requirements, would be required to fully inform the feasibility of adapting this model more broadly.

There are several potential improvements that could be made to the Liver Toolkit to streamline future use. In this pilot study, the recall list was generated by project staff, and patients were flagged and subsequently recalled for assessment. The project took over 200 hours of direct staff time, equating to 1.25 hours per patient successfully recalled. A more automated system that used real-time “pop-up” notifications, could potentially identify patients at increased risk of ACLD when they were seeing their GP for other reasons. This would eliminate the need to recall patients separately, reduce overall time requirements, and potentially improve the number of patients reached. Such an approach has been trialed recently, and it found 1 in 3 patients were successfully linked to fibrosis assessment.^27^ This Liver Toolkit evaluated several NITs; from our data, the simpler tests (APRI and FIB-4) had better reach than the more complex NFS (which incorporates body mass index and diabetes status), as these additional factors were not always available in primary care records. This highlights that the higher potential diagnostic accuracy of an NIT must be balanced against test complexity when selecting the optimal NIT for community screening. Although we did not find a significant difference in the PPV for ACLD between APRI and FIB-4, using a single NIT would make the project logistically easier. FIB-4 has also now been recommended as the first-line NIT by several societies.^17,35^ Recalling patients only once an NIT was positive on 2 occasions would also increase the PPV of the tool. Finally, limiting the target population to adults between 40 and 75 years of age would reduce the number of patients needing recall to detect 1 case of ACLD while maximizing yield, as most cases of new ACLD were found in this age range.

## CONCLUSIONS

This pilot of a novel information technology system (the Liver Toolkit) successfully screened a large primary care cohort to identify patients at increased risk of ACLD. More than 1 in 5 patients recalled were found to have significant liver disease needing ongoing follow-up. Major limitations identified were the low response rate to recall and the low PPV of the tool. Further work is needed to improve the overall utility to make more widespread population screening feasible.

## Supplementary Material

SUPPLEMENTARY MATERIAL
