# Supplementary material for: Screening patients in general practice for advanced chronic liver disease using an innovative IT solution: The Liver Toolkit
Source: Hepatol Commun. 2024 Jun 27;8(7):e0482. doi: 10.1097/HC9.0000000000000482 (PMC11213595; doi:10.1097/HC9.0000000000000482)
Supplement: SUPPLEMENTARY MATERIAL [file hc9-8-e0482-s001.docx]

# Supplementary Appendix

Supplementary figure 1 – Example Liver Toolkit patient recall list based on simulated patient data


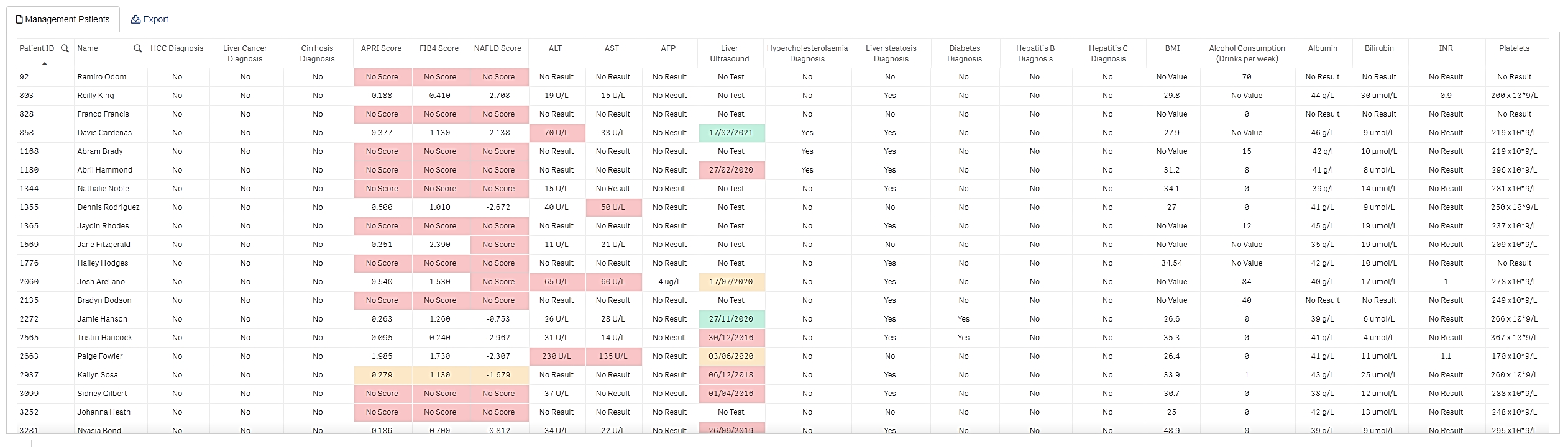


Supplementary Table 1 – Performance of repeat APRI and FIB-4 testing in patients with initially elevated results

| NIT | NIT normalised on repeat testing  n(%) | ACLD detection in patients with a persistently elevated NIT  n(%) | ACLD detection in patients who NIT normalised on repeat testing  n(%) | P value † |
| --- | --- | --- | --- | --- |
| High APRI score (≥1.0) (n=42) | 14/42 (33%) | 12/28 (43%) | 0/14 (0%) | **0.003** |
| High FIB-4 score (≥3.25) (n=33) | 11/33 (33%) | 9/22 (40%) | 3/11 (27%) | 0.7026 |

Abbreviations: ACLD – Advanced chronic liver disease, APRI – Aspartate aminotransferase to platelet ratio index, FIB-4 – Fibrosis 4 score, NIT – Non-invasive test

† Fisher’s exact test
